# Supplementary material for: Representation and detection of inversions in pangenome graphs
Source: NAR Genom Bioinform. 2026 Oct 15;8(4):lqag125. doi: 10.1093/nargab/lqag125 (PMC13669635; doi:10.1093/nargab/lqag125)
Supplement: Supplementary file 1 [file lqag125_supplemental_file.pdf]

Supplementary material for
Representation and detection of inversions in pangenome graphs

Sandra Romain[1,4], Siegfried Dubois[1,2], Fabrice Legeai[1,3], and Claire Lemaitre[1]

[1] *Univ Rennes, Inria, CNRS, IRISA - UMR 6074, F-35000 Rennes, France*
[2] *GenPhySE, Université de Toulouse, INRAE, ENVT, 31326 Castanet Tolosan, France*
[3] *IGEPP, INRAE, Institut Agro, University of Rennes, 35653 Le Rheu, France*
[4] *Current affiliation: Bart's Cancer Institute, Queen Mary University London, EC1M 6AU London, UK*

1 Supplementary material and methods

1.1 Command lines for the different PG pipelines

Minigraph pipeline

```
# graph construction
minigraph -cxggs refId.fa simulated.fa > minigraph.gfa

# List graph bubbles
gfatools bubble minigraph.gfa > gfatools.bed

# Extract haplotype(s) path in bubbles
    #for each haplotype (including the reference)
    minigraph -cxasm --call -t8 minigraph.gfa hapId.fa > hapId-path.bed

ls *-path.bed > samples.txt
paste *-path.bed | mgutils.js merge -s samples.txt - > merged.bed

mgutils-es6.js merge2vcf -r0 merged.bed > minigraph-bubbles.vcf

# bubble annotation
invpg -v minigraph-bubbles.vcf -g minigraph.gfa -o minigraph_inversions.vcf -m 0.5 -d 10
```

Minigraph-Cactus pipeline

```
# Create the pipeline.txt file containing on each line, the haplotype ID
# and the full path of the corresponding sequence fasta file (tab separated)

#Graph construction
JB=jobstore/js.0
cactus-minigraph $JB pipeline.txt CACTUS/graph.gfa --reference refId \
        --binariesMode singularity
cactus-graphmap $JB pipeline.txt CACTUS/graph.gfa CACTUS/graph.paf  --reference refId \
        --outputFasta CACTUS/graph.sv.gfa.fa.gz --binariesMode singularity
cactus-align $JB pipeline.txt CACTUS/graph.paf CACTUS/graph.hal --pangenome --outGFA \
        --outVG --reference refId --workDir CACTUS/workdir --binariesMode singularity
cactus-graphmap-join $JB --vg CACTUS/graph.vg --outDir CACTUS/outdir --outName final \
        --reference refId --clip 0 --filter 0 --binariesMode singularity

gzip -d CACTUS/outdir/final.full.gfa.gz
```

```
# Convert the graph in GFA1.1 to GFA1.0 format with all P-lines
vg convert -g -f -W CACTUS/outdir/final.full.gfa > mgc.gfa

# bubble calling
vg deconstruct -a mgc.gfa -p refId > mgc-bubbles.vcf

# bubble annotation
invpg -v mgc-bubbles.vcf -g mgc.gfa -o mgc_inversions.vcf -m 0.5 -d 10
```

PGGB pipeline

```
# prepare input haplotypes
cat haplotype1.fa haplotype2.fa > all_haplotypes.fa

# graph construction
pggb -i all_haplotypes.fa -o outdir -n 2 -t 8 -p 90 -s 5k -V refId
mv outdir/all_haplotypes.fa.*.smooth.final.gfa pggb.gfa

# bubble calling
vg deconstruct -a pggb.gfa -p refId > pggb-bubbles.vcf

# bubble annotation
invpg -v pggb-bubbles.vcf -g pggb.gfa -o pggb_inversions.vcf -m 0.5 -d 10
```

Cactus pipeline

```
# Create the input_cactus.txt file
# First line of this file = binarized genome tree
# Then one line per haplotype, with its ID and the absolute path of the corresponding
# sequence fasta file (tab separated)
    Eg.
    (refId:1.0,simulated:1.0);
    refId   /path/to/refId.fa
    simulated   /path/to/simulated.fa

#Graph construction
cactus --binariesMode singularity ./js input_cactus.txt cactus.hal
hal2vg --noAncestors --refGenomes refId cactus.hal > cactus.pg
vg convert --gfa-out -W cactus.pg > cactus.gfa

# bubble calling
vg deconstruct -a cactus.gfa -p refId > cactus-bubbles.vcf

# bubble annotation
invpg -v cactus-bubbles.vcf -g cactus.gfa -o cactus_inversions.vcf -m 0.5 -d 10
```

2 Supplementary tables

2.1 Details on inversion annotations for the different PG pipelines on the 2-haplotypes simulated datasets

Supplementary Table 1: Inversion bubble statistics of the pangenome graphs obtained for the 2-haplotype simulated genome sets with 100 inversions and with varying percentages of punctual polymorphism, for each pangenome graph constructing tool used. The number of inversion bubbles indicates the total number of bubbles annotated as inversions by our method. The "Non redundant and TP bubbles" column indicates the number of inversion bubbles that do not overlap any other inversion bubble and that are True Positive bubbles (ie. with at least 50 % reciprocal overlap with a simulated inversion). The "Redundant bubbles" column indicates the number of inversion bubbles that overlap at least one other inversion bubble and all overlap a simulated inversion by at least one bp. "Imprecise" bubbles indicate the number of inversion bubble that overlap a simulated inversion by less than 50 % (not counted in the Recall). The Recall column indicates the percentage of simulated inversions (among the 100) that have a significant reciprocal overlap with at least one inversion annotated bubble. ("MC": Minigraph-Cactus).

Genome set	PG pipeline	Total bubbles (n)	Inversion bubbles	Non redundant TP bubbles (n)	Redundant bubbles (n)	Imprecise bubbles (n)	Recall (%)
0% SNP	Cactus	766	92	92	0	0	92
	Minigraph	21	7	4	0	3	4
	MC	2,680	28	16	2	5	16
	PGGB	333	92	90	0	2	90
0.1% SNP	Cactus	28,735	92	92	0	0	92
	Minigraph	82	75	72	0	3	72
	MC	121,368	90	78	0	4	78
	PGGB	28,233	90	89	0	1	89
1% SNP	Cactus	277,961	92	92	0	0	92
	Minigraph	75	75	75	0	0	75
	MC	322,643	92	82	0	2	82
	PGGB	275,670	90	89	0	1	89
5% SNP	Cactus	1,330,968	91	67	24	1	90
	Minigraph	74	73	73	0	0	73
	MC	1,201,768	85	79	0	3	79
	PGGB	1,312,279	89	89	0	0	89

2.2 Details on inversion annotations for the different PG pipelines on the 10-haplotypes simulated datasets

Supplementary Table 2: Inversion bubble statistics of the pangenome graphs obtained for the 10-haplotype simulated genome sets with 100 inversions and with 1% of punctual polymorphism, for each pangenome graph constructing tool used. The number of inversion bubbles indicates the total number of bubbles annotated as inversions by our method. The "Non redundant TP bubbles" column indicates the number of inversion bubbles that do not overlap any other inversion bubble and that are True Positive bubbles (ie. with at least 50 % reciprocal overlap with a simulated inversion). The "Redundant bubbles" column indicates the number of inversion bubbles that overlap at least one other inversion bubble and all overlap a simulated inversion by at least one bp. "Imprecise" bubbles indicate the number of inversion bubble that overlap a simulated inversion by less than 50 % (not counted in the Recall). The Recall column indicates the percentage of simulated inversions (among the 100) that have a significant reciprocal overlap with at least one inversion annotated bubble. ("MC": Minigraph-Cactus).

Genome set	PG pipeline	# Inversion bubbles	# Non redundant TP bubbles	# Redundant bubbles	# Imprecise bubbles	Recall (%)
1% SNP	Cactus	162	30	132	1	90
	Minigraph	76	76	0	0	76
	MC	88	85	2	8	86
	PGGB	94	92	0	2	92

2.3 Performances of INVPG_annot on the real human pangenome graphs

Supplementary Table 3: Cumulated graph size, total number of bubbles and running time of INVPG_annot on the human chromsome 7 and chromosome X pangenome graphs produced by the 4 pipelines. INVPG_annot was run on a single core of Intel(R) Xeon(R) CPU E5-2630 v4 @ 2.20GHz. "MC": Minigraph-Cactus.

PG pipeline	Graph size (Mbp)	# Bubbles	Running time (min)	RAM (Mb)
Cactus	343	1,512,364	8min07s	669
Minigraph	319	4,620	2min12s	174
MC	371	1,042,944	14min08s	442
PGGB	310	1,024,316	35min11s	535

3 Supplementary figures

3.1 Impact of INVPG-annot parameters on recall values

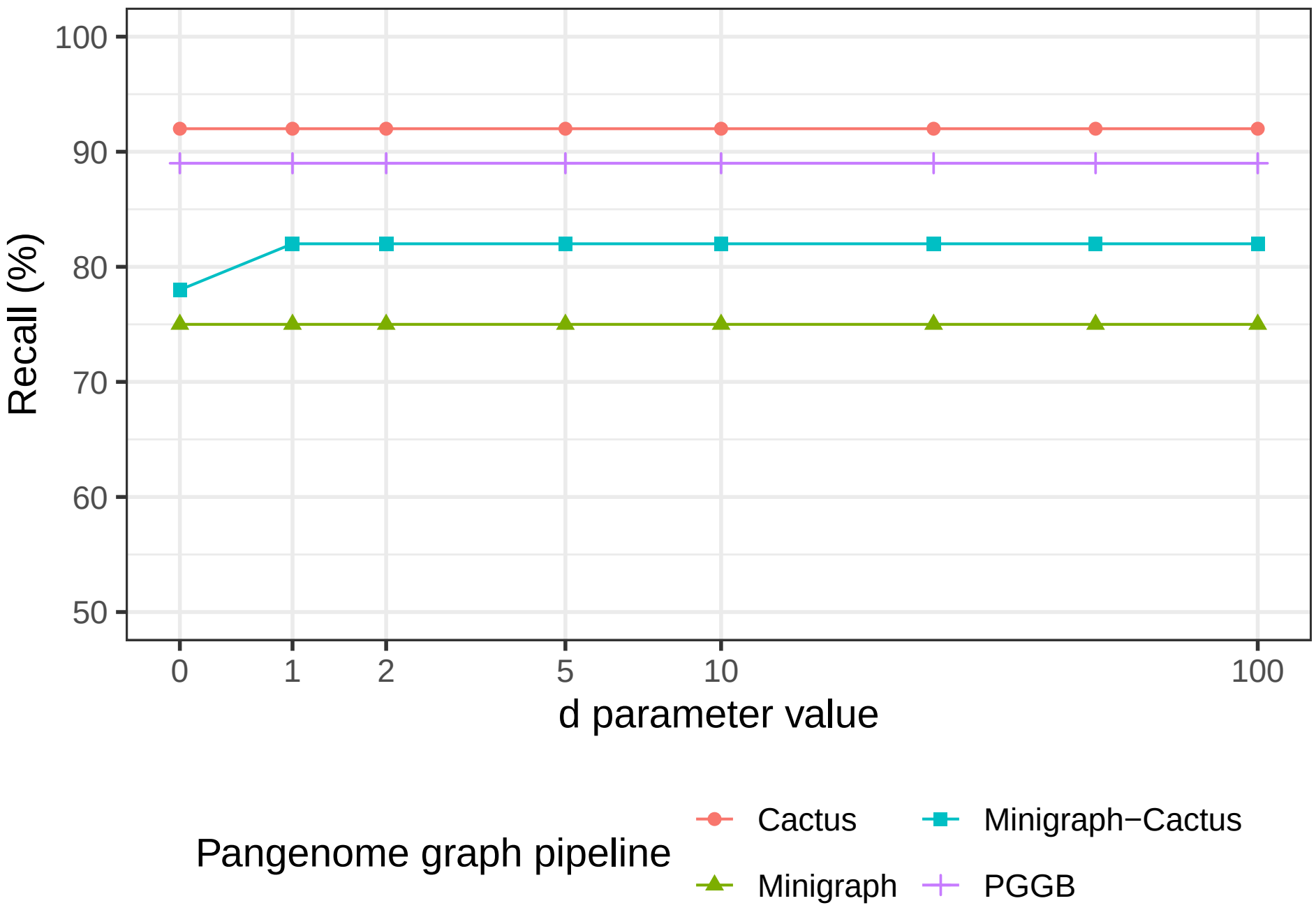

Supplementary Figure 1: Effect of the maximum allowed size divergence between allele lengths (parameter -d) on inversion recall, evaluated on the 2-haplotype graphs constructed from simulated haplotypes with 1% nucleotide polymorphism and 100 inversions. The parameter -m was set to its default value of 0.5.

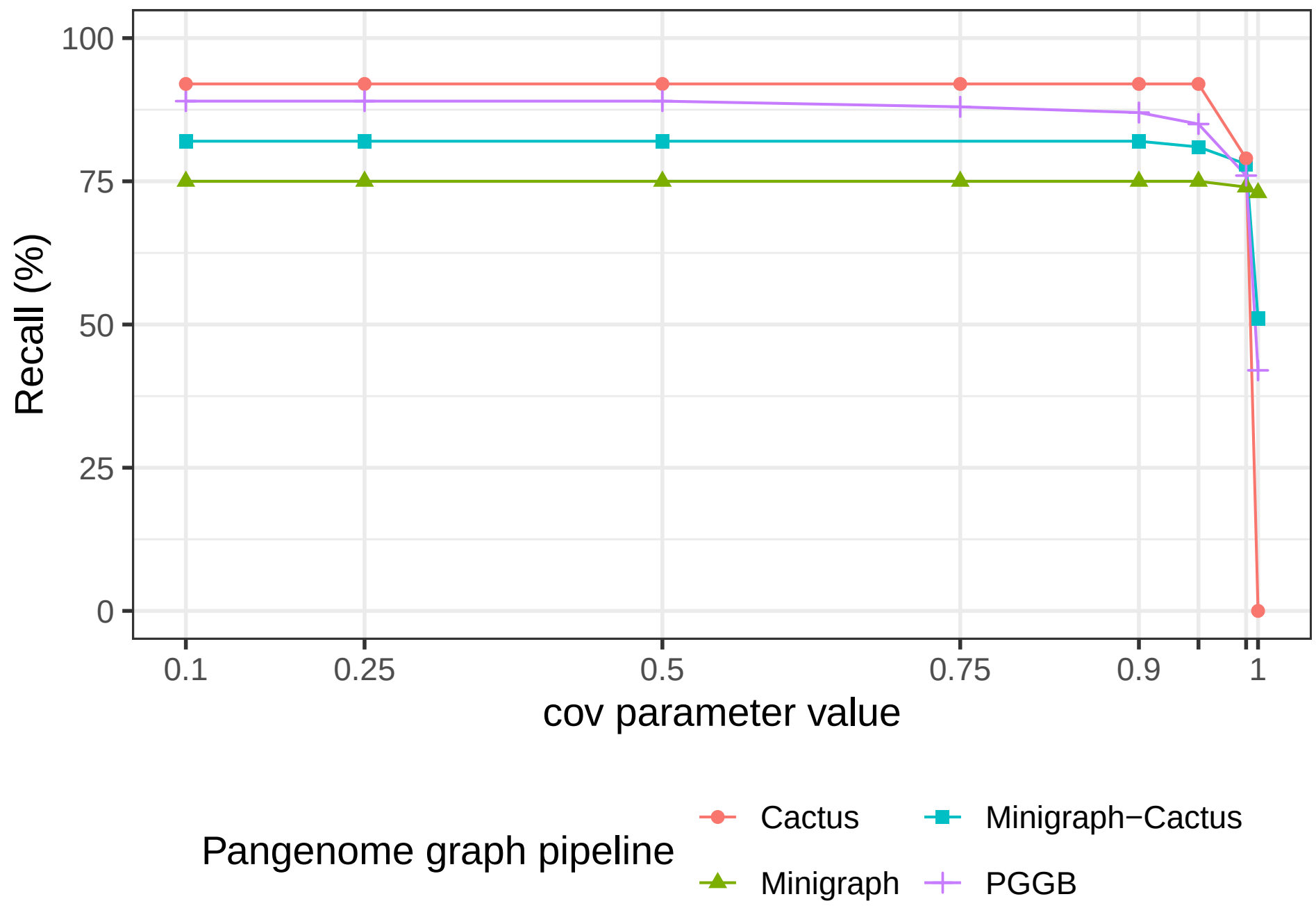

Supplementary Figure 2: Effect of the minimum inversion signal coverage threshold (parameter -m) on inversion recall, evaluated on the 2-haplotype graphs constructed from simulated haplotypes with 1% nucleotide polymorphism and 100 inversions. The two *cov* values not displayed on the figure are 0.95 and 0.99. The parameter -d was set to its default value of 10.

3.2 Impact of minimum overlap requirement on recall values

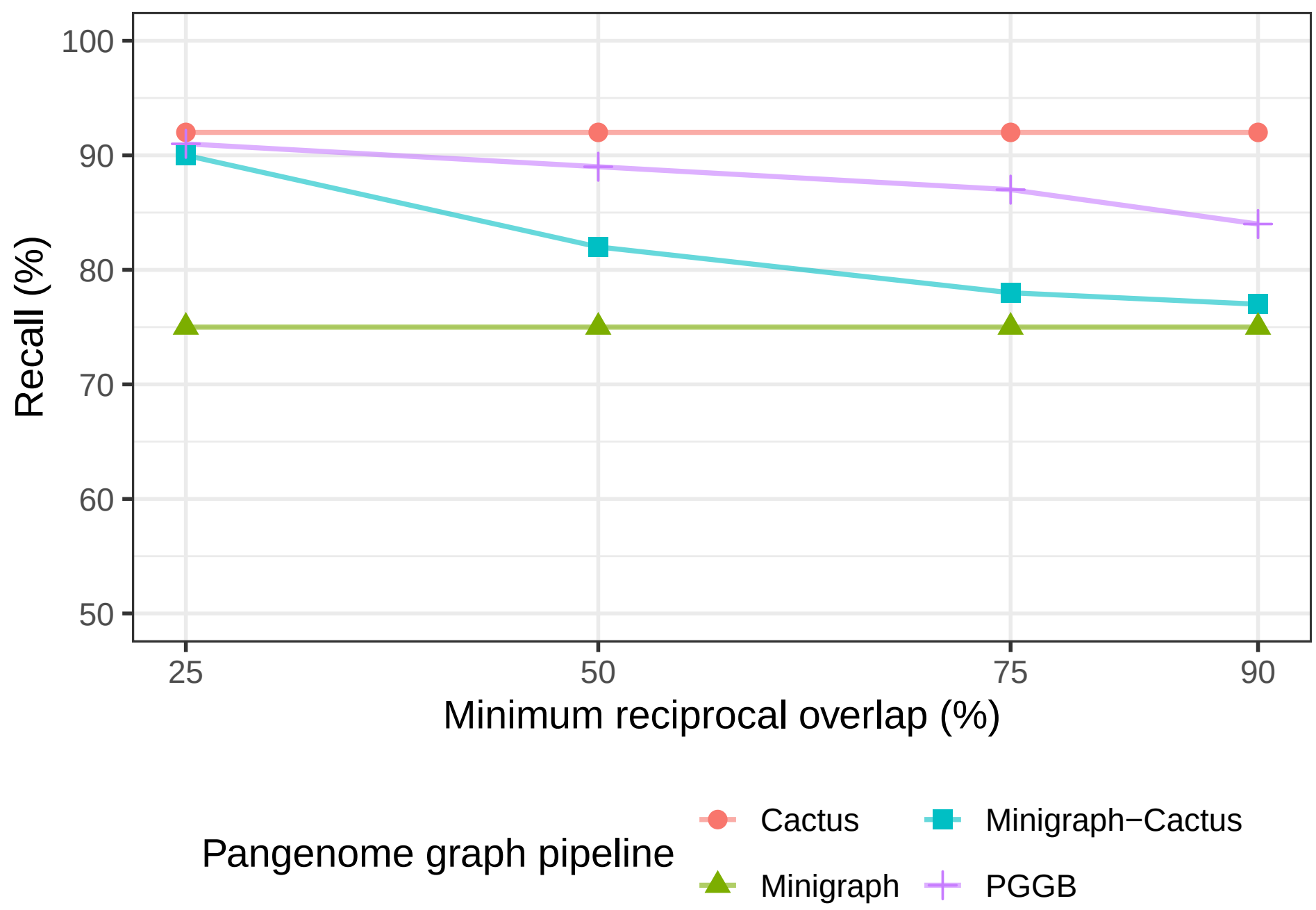

Supplementary Figure 3: Effect of the minimum reciprocal overlap threshold required to count a true positive for inversion recall, evaluated on the 2-haplotype graphs constructed from simulated haplotypes with 1% nucleotide polymorphism and 100 inversions. Parameters -m and -d were set to their default values of 0.5 and 10, respectively.

3.3 Graph visualisation examples at missed inversion loci

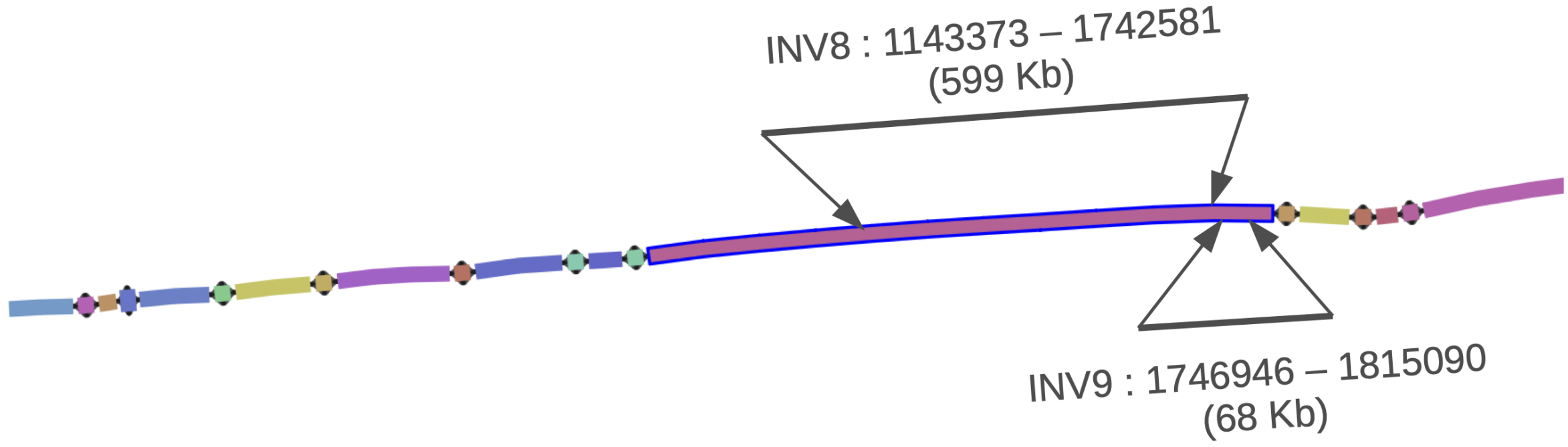

Supplementary Figure 4: Visualisation of a region of the Minigraph graph constructed from simulated dataset with 2 haplotypes with 1% nucleotide polymorphism and 100 inversions. The visualization was obtained with the tool Bandage (Wick et al., 2015). Node s15 (in pink) spans two loci of inversions, whose start and end positions are indicated by grey arrows. These inversions are not represented as graph variation and were therefore missed during graph construction.

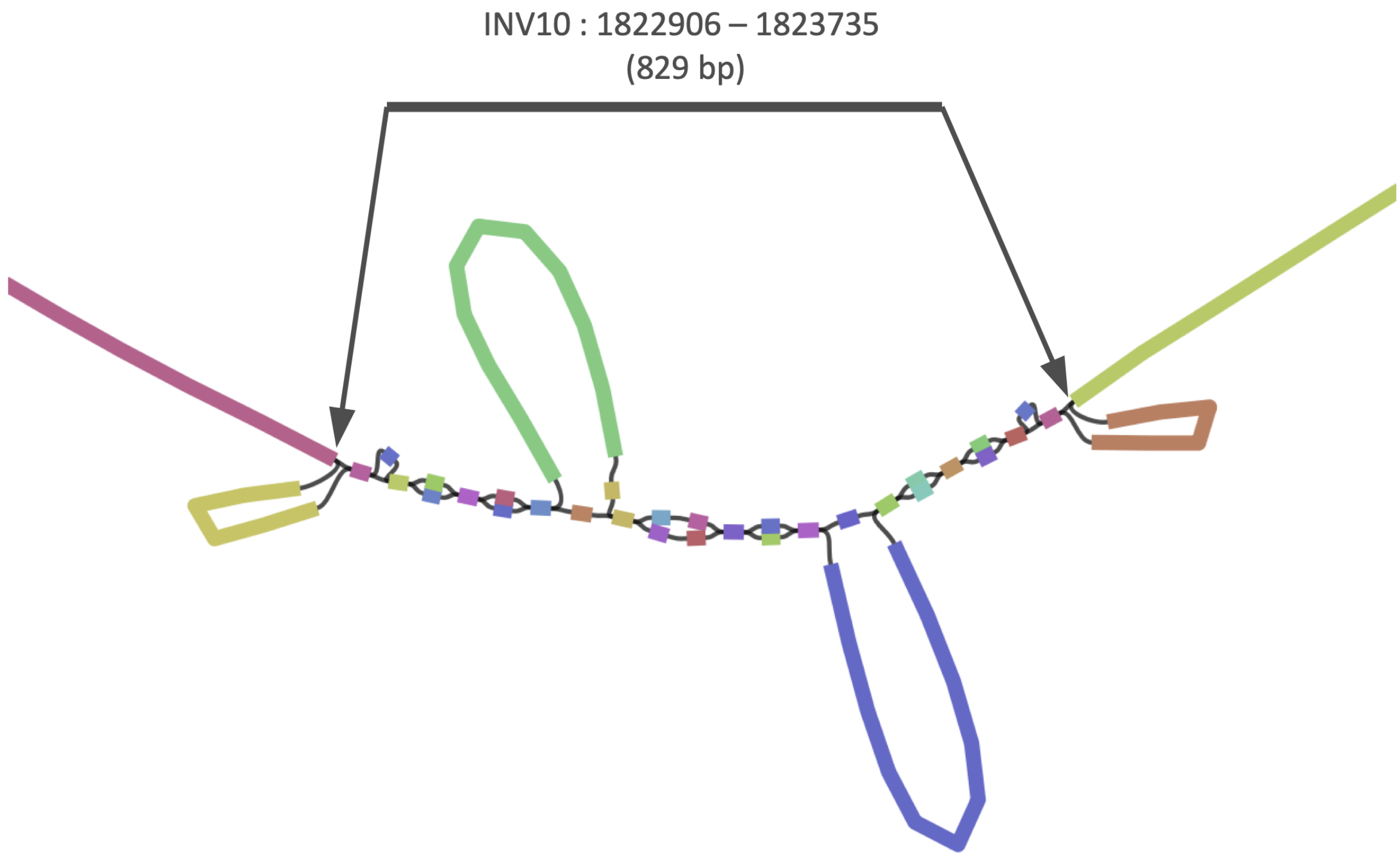

Supplementary Figure 5: Visualisation of a region of the Cactus graph constructed from simulated dataset with 2 haplotypes with 0.1% nucleotide polymorphism and 100 inversions. The visualization was obtained with the tool Bandage, where nodes are colored with different colours. Grey arrows indicate the start and end positions of a missed inversion of 829 bp. Between these two positions, instead of one inversion bubble, the graph contains 12 smaller bubbles, including two pairs of insertions and deletions of 250 and 523 bp.

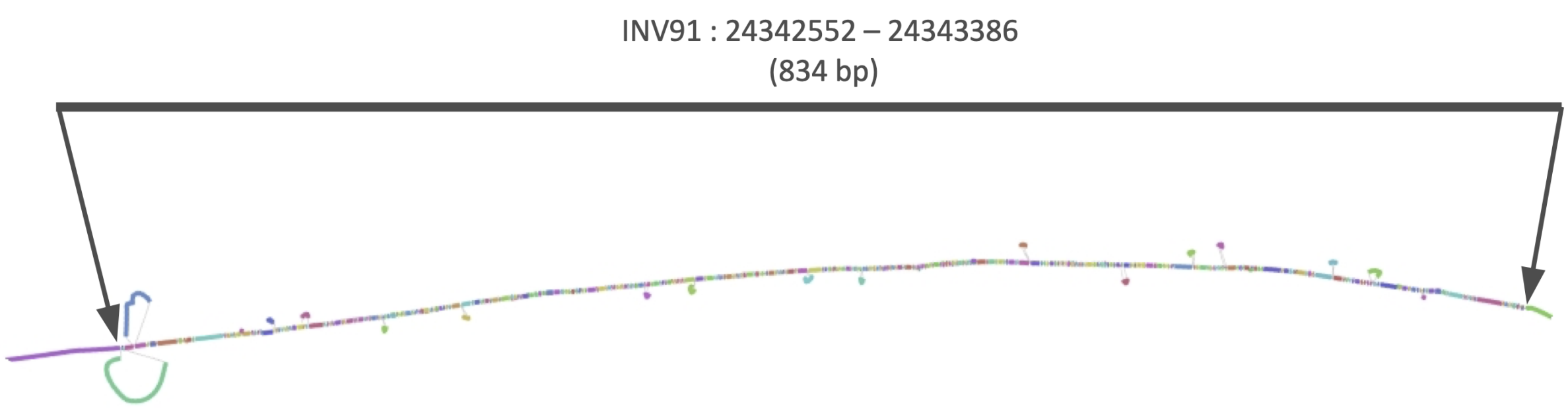

Supplementary Figure 6: Visualisation of a region of the Cactus graph, constructed from simulated dataset with 2 haplotypes with 0.1% nucleotide polymorphism and 100 inversions. The visualization was obtained with the tool Bandage, where nodes are colored with different colours. Grey arrows indicate the start and end positions of a missed inversion of 834 bp. Between these two positions, instead of one inversion bubble, the graph contains 159 smaller bubbles, almost all of them smaller than 10 bp, suggesting that the alignment was forced in the forward orientation.

3.4 Example of redundant inversion bubbles

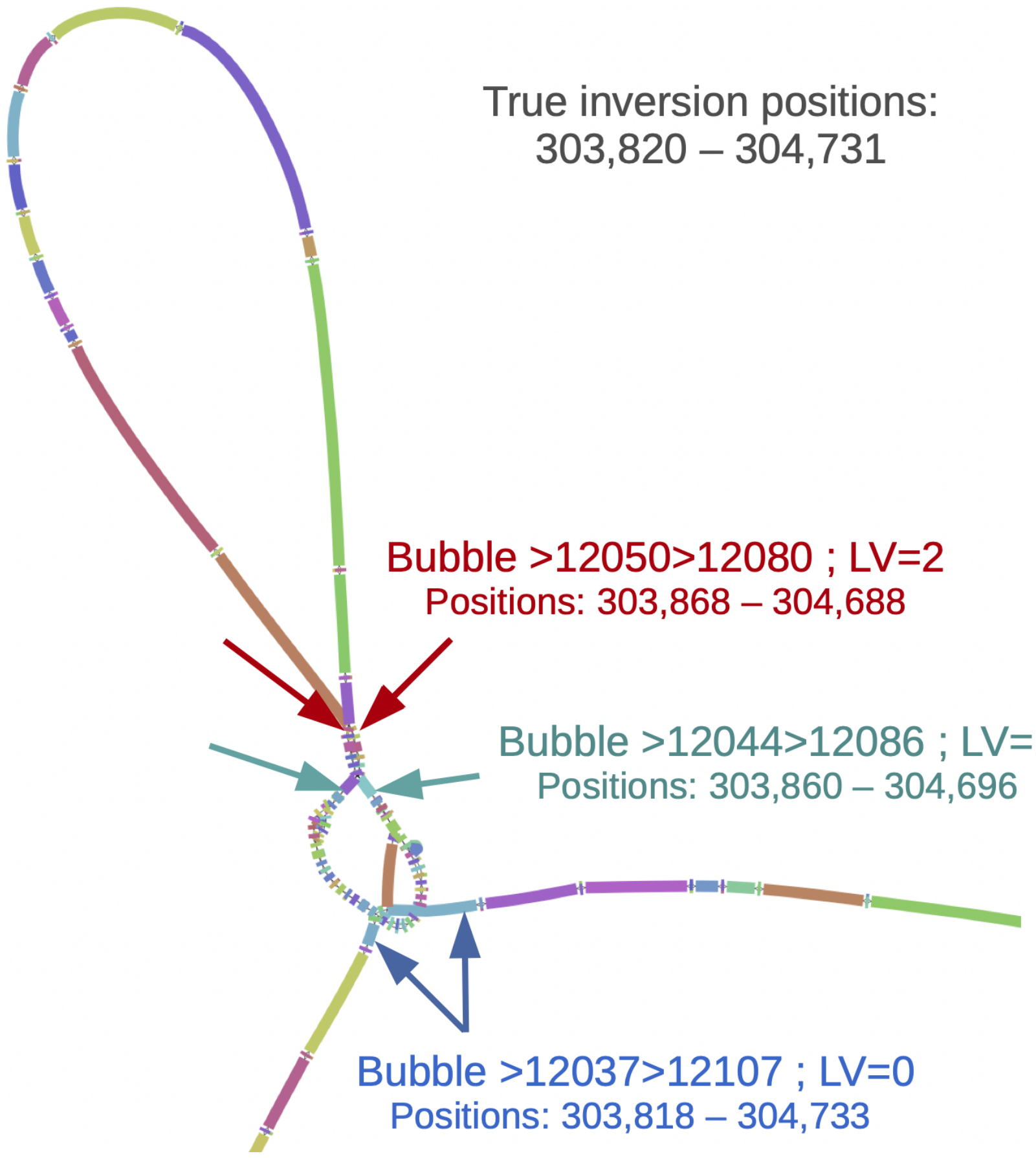

Supplementary Figure 7: Example of three redundant inversion bubbles in the Cactus graph constructed from simulated dataset with 10 haplotypes with 1% nucleotide polymorphism and 100 inversions. The visualization was obtained with the tool Bandage. Pairs of arrows of the same colour indicate the source and sink nodes of each bubble. The three bubbles are nested within one another (LV denotes the nesting level, with 0 corresponding to the outermost bubble), and each of these three bubbles overlaps the same true inversion locus with more than 50% reciprocal overlap.

References

R. R. Wick, M. B. Schultz, J. Zobel, and K. E. Holt. Bandage: interactive visualization of de novo genome assemblies. *Bioinformatics*, 31(20):3350–3352, 2015. doi: 10.1093/bioinformatics/btv383.
